# Development of a Multimodal Apparatus to Generate Biomechanically Reproducible Spinal Cord Injuries in Large Animals

**DOI:** 10.3389/fneur.2019.00223

**Published:** 2019-03-19

**Authors:** Mark Züchner, Andreas Lervik, Elena Kondratskaya, Vanessa Bettembourg, Lili Zhang, Henning A. Haga, Jean-Luc Boulland

**Affiliations:** ^1^Department of Neurosurgery, Oslo University Hospital, Oslo, Norway; ^2^Norwegian Center for Stem Cell Research, Oslo University Hospital, Oslo, Norway; ^3^Department of Companion Animal Clinical Sciences, Norwegian University of Life Sciences, Oslo, Norway; ^4^Institute for Experimental Medical Research, Oslo University Hospital and University of Oslo, Oslo, Norway

**Keywords:** spinal cord injury, apparatus, pig, testing therapies, impactor

## Abstract

Rodents are widespread animal models in spinal cord injury (SCI) research. They have contributed to obtaining important information. However, some treatments only tested in rodents did not prove efficient in clinical trials. This is probably a result of significant differences in the physiology, anatomy, and complexity between humans and rodents. To bridge this gap in a better way, a few research groups use pig models for SCI. Here we report the development of an apparatus to perform biomechanically reproducible SCI in large animals, including pigs. We present the iterative process of engineering, starting with a weight-drop system to ultimately produce a spring-load impactor. This device allows a graded combination of a contusion and a compression injury. We further engineered a device to entrap the spinal cord and prevent it from escaping at the moment of the impact. In addition, it provides identical resistance around the cord, thereby, optimizing the inter-animal reproducibility. We also present other tools to straighten the vertebral column and to ease the surgery. Sensors mounted on the impactor provide information to assess the inter-animal reproducibility of the impacts. Further evaluation of the injury strength using neurophysiological recordings, MRI scans, and histology shows consistency between impacts. We conclude that this apparatus provides biomechanically reproducible spinal cord injuries in pigs.

## Introduction

SCI often results in devastating neurological deficits with a major impact on the quality of life. The worldwide incidence of traumatic SCI varies substantially between different reports: It ranges from 3.6 to 1,009 patients per one million inhabitants (1, 2). Moreover, SCI is not limited to traumatic patients; it may arise from other etiologies such as degenerative diseases of the vertebral column, tumors, and infections (3). As a result of progress in rehabilitation, SCI patients benefit from a longer life expectancy. As a consequence, SCI has become a public health problem, while the financial burden for society caused by the cost of care and productivity loss is immense (4, 5). Treatments that improve the function clearly have a major influence on the quality of life of patients. Moreover, they would be beneficial for society. It is, therefore, crucial to translate the basic research findings into clinical practice.

In the last few decades, the scientific community has exerted substantial efforts to overcome the consequences of SCI. An increasing number of promising therapeutic approaches have been developed in animal models, predominately in rats or mice (6, 7). Rodents present many advantages. They have been widely used in many different research areas. Hence, there is an immense literature spanning from molecular to behavioral approaches. Transgenic mice represent an invaluable tool to understand basic mechanisms that may be targeted to promote functional recovery. Rodents are also inexpensive and easy to handle; their housing does not require complicated logistics; and their use for SCI research is already widely established. Multiple methods, such as contusion, compression, and hemisection, have been developed to produce a controlled and reproducible injury (6). Several impacting device, used in different laboratories have been shown to produce reliable and graded injuries (8–11). In addition, behavioral, histological, and biochemical outcome measurements are well established in multiple laboratories (6, 12). However, many new experimental treatments that have shown efficacy in small animal models were difficult to translate in human clinical trials (13, 14). This discrepancy is probably a result of differences in the neuroanatomy, neurophysiology, and immune systems (15). In addition, the size of the animal is a conspicuous factor, particularly with respect to re-growing axons. One millimeter of growth may be significant in rodents, but insufficient in larger animals or humans. The thickness of the spinal cord and the volume of cerebrospinal fluid (CSF) are also important factors that may influence medication-based therapies for which dilution and penetration of the chemical into the tissue is critical. So, SCI research tends to evolve toward animals of a higher order which are closer to human anatomy and physiology (15–17).

Since there is no commercially available impactor to generate SCI in large animals, we describe the development of an apparatus to perform biomechanically reproducible SCI in pigs weighing 25–50 kg. We show the iterative steps in this development, particularly focusing on the reliability and versatility of the contusion/compression device and the different surgical aspects. The multimodal sensors, the electrophysiology recordings, and the postoperative and post-mortem analyses show that our system generates reproducible acute injuries. The development of a reliable apparatus and the implementation of a surgical protocol is an essential step toward the establishment of a completely characterized animal model.

## Materials and Methods

### Study Overview

We developed two different impacting systems. During Phase 1 of the study, we produced a weight-drop impactor inspired by the early work of Allen (18), and its more recent developed version adapted to rodents and minipigs (19–25). For Phase 2, we developed a spring-load impactor which, to our knowledge, represents a novel system. Both systems were tested on different artificial surfaces before the initiation of *in vivo* experiments to determine the reproducibility of impacts. In complement to the spring-load impactor we developed tools to ensure reproducibility of the impacts and to ease the surgery.

### Animals

The animal experiments were approved by the Norwegian Animal Research Authority (Forsøksdyrutvalget, FDU ID. 7089). The FELASA guidelines were followed. In compliance with these regulations, all efforts were made to minimize the number of animals used and their suffering. All surgical procedures were supervised by veterinarians. The experiments were performed on 20 male or female domestic pigs that weighed between 25 and 50 kg (mean: 28.7 kg; median: 26 kg) The pigs used for this study had a genetic background composed of 50% Norwegian land pig, 25% Norwegian Yorkshire, and 25% Duroc. In addition, seven pig cadavers were used to test the instrument prototypes.

### Engineering of Instruments

All instruments engineered for this study were produced at a local workshop facility. The sketching was performed using the 3D CAD design software (Solidworks, Dassault Systèmes, Waltham, MA, USA).

**Figure 1:**
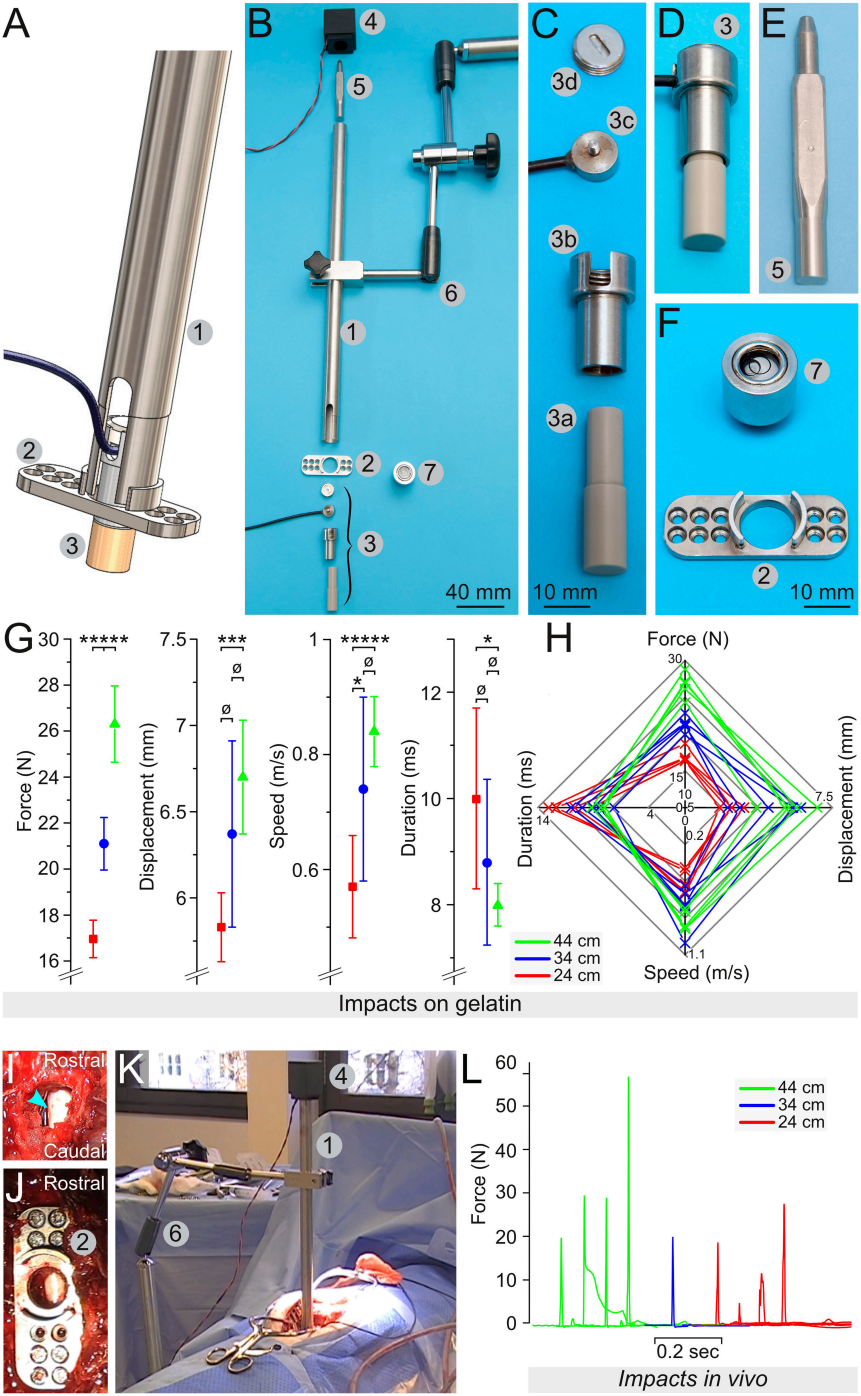
Engineering of a weight-drop spinal cord impactor. **(A)** Computer model showing the assembly of the impactor with the pipe (1), the lamina plate (2), and the plunger (3). **(B)** All elements composing the impactor: the pipe (1), the lamina plate (2), the plunger (3), the electromagnet (4), the 50 g weight (5), the articulated arm (6), and the spirit level (7). **(C)** The plunger is an assembly of the plunger tip (3a), the sensor chamber (3b), the load button-sensor (3c), and the screw cap that closes the sensor chamber (3d). **(D)** The plunger assembled. **(E)** The 50 g weight shaped to reduce friction. **(F)** The lamina plate (2) to be attached on the lamina via small screws and a spirit level (7) that fits the hole of the plate, as well as the opening at the top of the pipe. **(G)** Mean values for the force, the displacement, the speed, and the duration recorded during test impacts on a 25-mm layer of 10% gelatin in PBS. The weight was dropped from pipes of different heights, including 24 cm (green), 34 cm (blue) and 44 cm (red). **(H)** Radar plots showing the relationship among the force, the displacement, the speed, and the duration for each impact. **(I–K)** Assembly of the impactor *in vivo*, with a dorsal laminectomy **(I)**, the fixation of the lamina plate **(J)**, and the positioning of the articulated arm (6) that supports the pipe (1) on the top of which the electromagnet (4) is fitted and holds the 50 g weight **(K)**. **(L)** Impact force recorded in 9 different pigs. Error bars represent the standard deviation. ø, *p* > 0.05; ^*^p < 0.05; ^***^p < 0.01; ^*****^p < 0.001.

For Phase 1, the weight-drop impactor is composed of guiding pipes of different lengths (Figures 1A,B), in which a 50-g weight released by an electromagnet falls onto a plunger. The weight was designed with four beveled edges to reduce contact-points and friction when gliding along the pipe (Figure 1E). The plunger contained a load button-sensor (model# LLB215, Futek, CA, USA) to measure the impact force and its dynamics (Figures 1C,D). The pipe was supported by an articulated arm (Mitutoyo Magnetic stand) and fitted to a plate affixed onto the laminae with screws (Figure 1F). The lamina plate was designed to fulfill three functions (1) fusion of adjacent levels (2) guidance of the plunger (3) attachment of the pipe. Further description of the assembly of this system is provided below.

**Figure 2:**
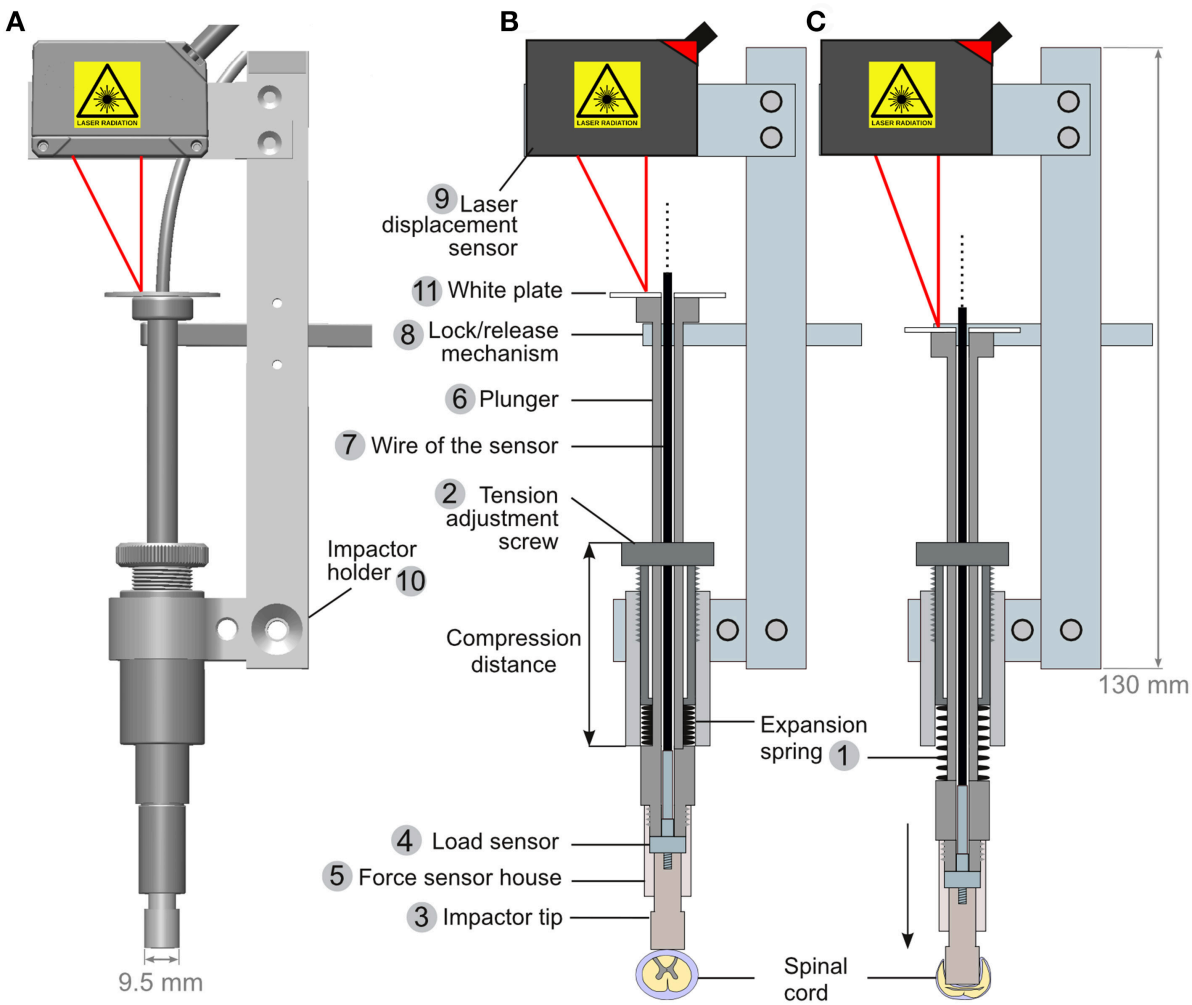
Engineering of a spring-load impactor. **(A)** Computer model of the impactor. **(B,C)** Drawings of a longitudinal section of the impactor when armed **(B)** and released **(C)**. The impactor is powered by the release of a compressed expansion spring (1). The compression of the spring is regulated by adjusting a screw (2). Turning on the screw increases the spring compression, and a stronger expansion is generated upon release. The impactor tip (3) is attached to a load button-sensor (4) and fitted in the force sensor house (5), itself screwed onto the plunger (6) around which the expansion spring is positioned (1). The wire of the load sensor (7) is passed through the plunger and further connected to an amplifier and a computer (not drawn). When armed **(B)**, the plunger is maintained in position by a lock/release mechanism (see also Figure 3). A laser displacement sensor (9) is attached to the impactor holder (10). It triangulates a laser light reflected by a white plate (11) positioned at the top of the plunger.

For Phase 2, the spring-load impactor (Figure 2A) is built around a piston powered by an expansion spring (model# C04200452250S, Sodemann Industrifjedre A/S Viby J, Denmark) compressed between the bottom edge of a screw (tension adjustment screw, Figure 2B, element 2) and the upper part of the piston element. This design provides the mechanical force to vigorously propel the piston upon the release of the locking mechanism (described bellow). A load button-sensor incorporated in the tip of the piston measures the impact force. In addition, a laser-displacement sensor (model# ILD1420CL1, Micro-Epsilon, ME, USA: measuring range of 25 mm at 2 kHz) measures the movement of the piston. Turning on the tension adjustment screw increases the compression of the spring; it provides more power during the release. In contrast, turning off the screw decreases the compression and the strength of the impact. Each screw turn corresponds to a 1-mm increase of the compression distance—this enables a precise adjustment of the spring compression. The piston is sufficiently long to move over several centimeters. However, the maximum distance required for full compression of the spinal cord is <1 cm. At this distance, the spring is not entirely expanded and thus it presses on the piston, thereby delivering static compression to the spinal cord (Figure 2C). It is possible after the impact to turn on/off the tension adjustment screw to the desired level of compression force. This may be adjusted in real time using the read-out from the sensors. The laser displacement sensor uses triangulation on a white reflective plate (Figure 2, element 11) to determine the position. Since the impactor tip is in direct contact with the dura, the surgeon has continuous visual control of its position.

**Figure 3:**
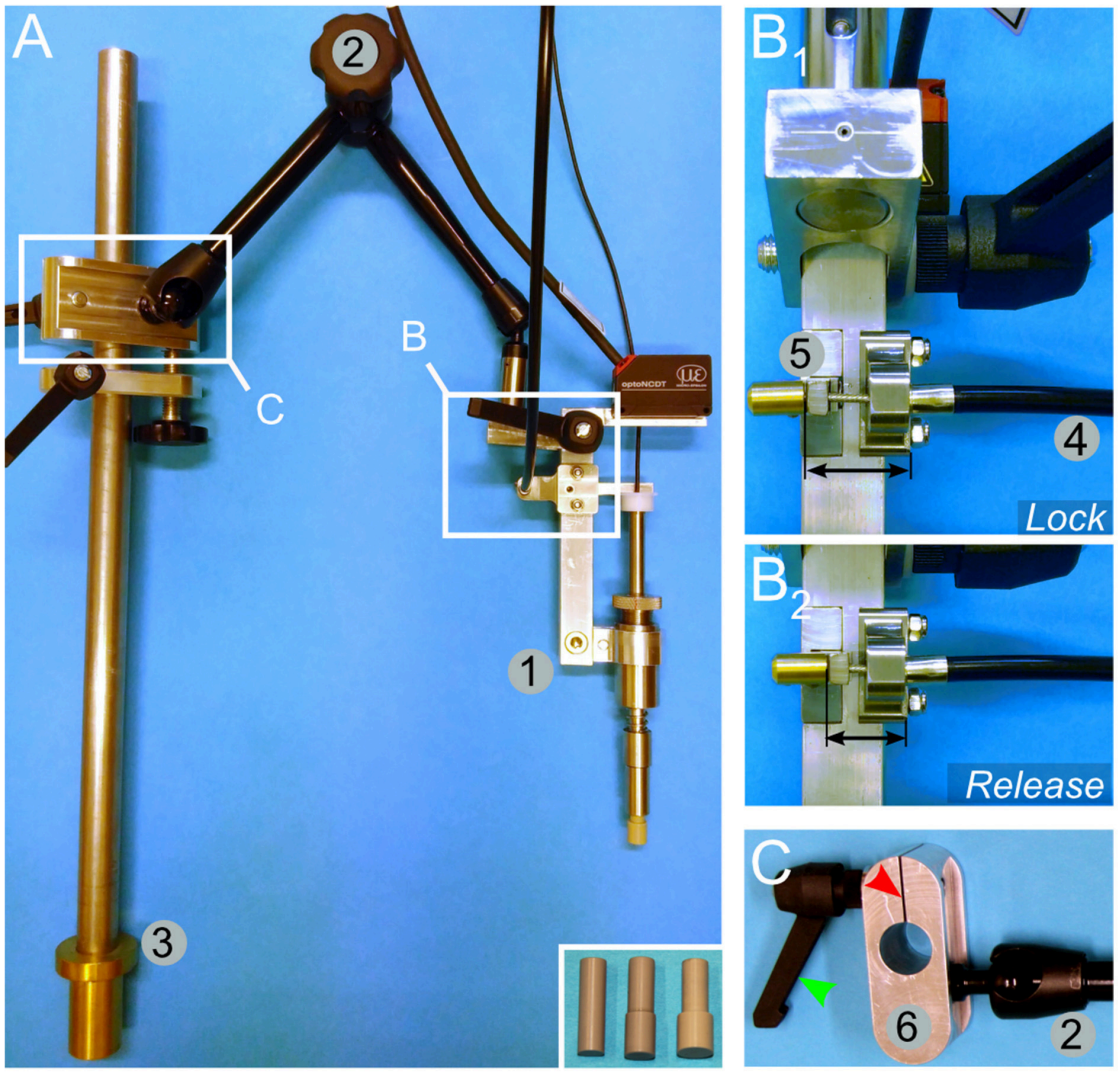
Mounting and engineering details for the spring load impacting system. **(A)** An overview image shows the impactor (1) mounted on an articulated arm (2) attached to a rod (3) by a sliding binder. The rod can be further fitted to the pig-operating table (Figure 5). The inset in **(A)** shows alternative impactor tips of different diameters. **(B)** Lock (B_1_)/release (B_2_) mechanism to trigger the impact. A bicycle wire (4) is attached to the lock/release bar (5). Pulling the wire moves the bar away from the impactor and thereby releases it. **(C)** Detailed view of the sliding binder (6). It is designed to be fitted to the rod shown in (**A**, element 3). The binder glides freely along the rod, but it can be locked in position by a screw handle (green arrowhead). Turning on the handle presses the cleft of the binder (red arrowhead) and reduces the diameter of the hole, thereby tightening its grip onto the rod.

The lock/release mechanism is performed through a stopper bar that keeps the piston in place when armed (Figure 2B, element 8). A bicycle wire (Figures 3A,B, element 4) was mounted on the stopper bar (Figure 3B, element 5). Pulling the wire moves the bar away from the piston and triggers the release of the impactor. A spring mounted at the base of the bar ensures that once released, it is kept away from the piston.

For positioning the impactor, we mounted it onto an articulated arm (Figure 3A, element 2). The arm was further connected to a sliding binder (Figures 3A,C) that can be fitted to a rod, which is attached to the operating table as described below.

For a better positioning of the pig, we produced an operating table inspired by previous descriptions (26, 27). It is designed to reduce the intra-abdominal pressure in the prone position to avoid extensive intra-spinal bleeding. The table (**Figure 5A**) contains two large rubber bands to support the pig. They are fitted on T-shaped elements. Their position may be adjusted in both the rostro-caudal and left-right axes by a system of rails and screw-locks (**Figures 5B,C**). If desired, they may be positioned further apart to leave the abdominal region free (**Figure 5E**)—this requires the use of additional lateral adjustable supports to avoid lordosis of the vertebral column (**Figures 5E,F**). They are composed of rods terminated by a large plastic disc to disperse the pressure. To avoid skin sores, gel pads are positioned between the disc and the skin of the pig. In a later stage of engineering, we replaced these lateral supports by a suspension system (further described below). Since the table is constructed of an aluminum bracket with rails, it enables the possibility to affix multiple elements, such as an articulated arm, to support the impactor.

The reproducibility of the biomechanical parameters of the impacts depends on several variables. In this study, we addressed four main factors: (1) the resistance of the surface underneath the spinal cord; (2) the capacity of the spinal cord to “escape” the impact by sliding to the side; (3) the absorption of energy by flexion of the spinal column during the impact; and (4) the angle between the piston and the spinal cord. To gain control over (1) and (2), we engineered a support system that encloses the ventral and lateral aspects of the spinal cord (**Figure 7**); it is composed of a link element (**Figures 7A,B**) mounted on a rod fitted onto pedicle screws. Two plates (one straight plate and one bended plate) are attached to the link and glided under the spinal cord to enclose it (**Figures 7C–E**). Flexion of the vertebral column during the impact is prevented by the fusion of three adjacent segments with pedicle screws and titanium rods (**Figure 7E**). To obtain a plain contact between the dura and the piston, we straightened the vertebral column with a suspension system (**Figure 6**). It is composed of a stand attached to the pig-operating table with screws (**Figures 6A–E**). Suspension rods terminated by a hook can be positioned and affixed to the stand by a locking system (**Figures 6C,D**). As detailed below, the suspension rods are hooked to surgical implants to exert a vertical pull on the vertebral column (**Figures 6F–H**).

### Test Impacts on Artificial Surface

We performed several tests on artificial surfaces that mimicked the firmness of the spinal cord to assess the reproducibility and strength of the impacts.

For Phase 1, we performed impacts on Petri dishes that contained a 25-mm thick layer of porcine gelatin, 10% (w/v) diluted in 0.1 M phosphate buffered saline (PBS). The load sensor measured the impact force, while the movement of the plunger was recorded at 5,630 Hz with a NX3-S1 high-speed video camera (IDT, Tallahassee, FL, USA) fitted with a macro objective (Micro-Nikkor 55 mm, 1:3.5, Nikon). For the analyses of these recordings, we used ImageJ (28) and the plug-in “Manual tracking” https://imagej.nih.gov/ij/plugins/track/Manual%20Tracking%20plugin.pdf. We obtained data on the displacement, speed, and duration of the impact (Figure 1). For each pipe, we performed five to eight impacts, each time on a new area of the gelatin with a maximum of three impacts per dish.

**Figure 4:**
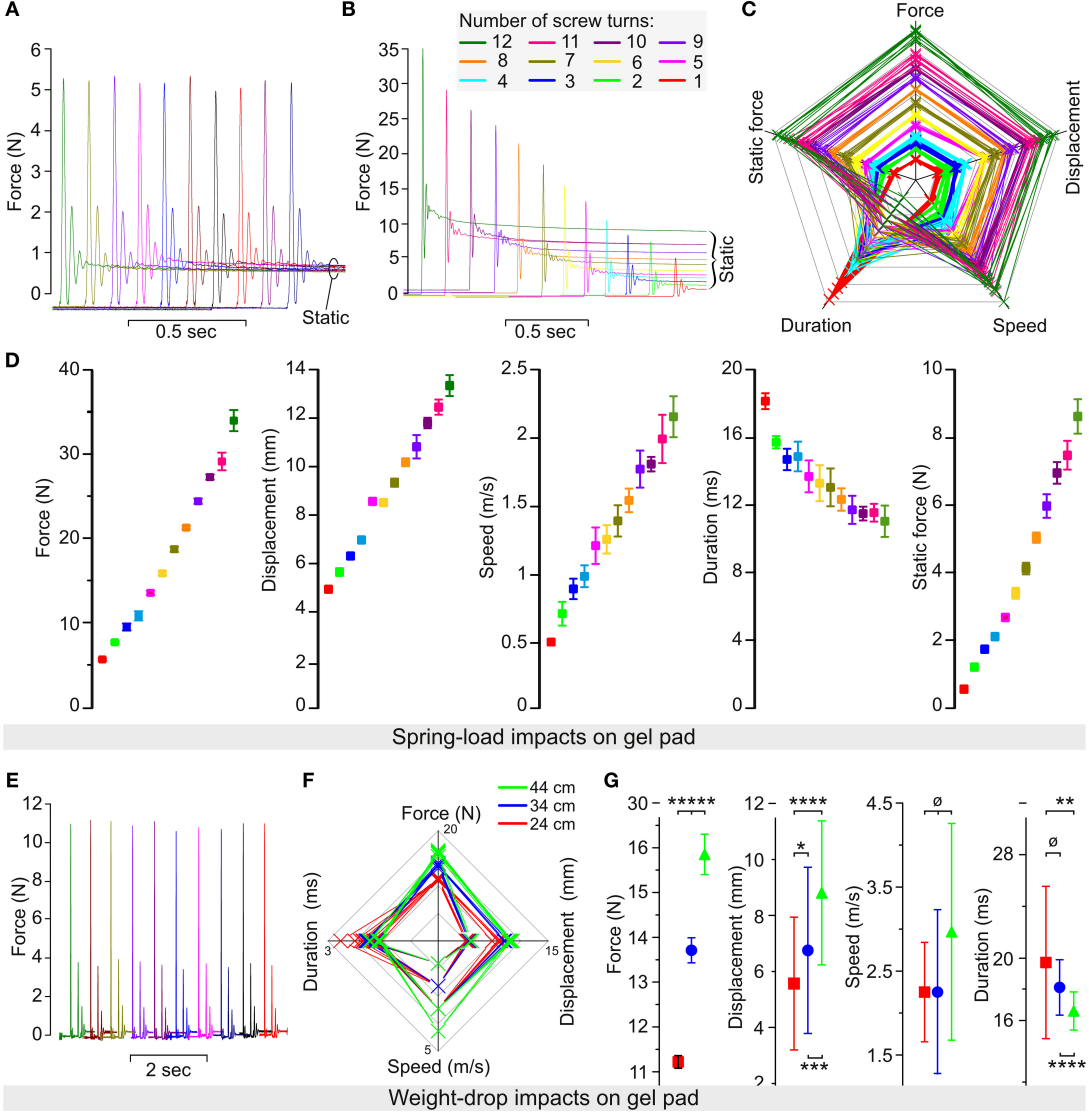
Reliability of the spring-load impactor tested on gel pad. **(A)** Force recorded during 10 impacts performed with the same setting for the expansion spring. **(B)** Force recorded during 12 impacts performed with a decreased number of screw turns corresponding to a reduction in the spring compression. **(C)** Radar plot showing the relationship among the force, the displacement, the speed, the duration, and the static force (post-impact) for each impact. Each color represents 10 impacts performed at a specific number of turns of the compression screw (from 1 to 12 turns). **(D)** Mean values for the force, the displacement, the speed, the duration, and the static force obtained from 10 impacts at each of the 12 different settings of the compression screw. Color coding is identical as in **(B,C)**. **(E–G)** Weight-drop test impacts performed on gel pads. The 50-g weight was dropped from pipes of different heights (10 trials per pipe), including 44-cm (green), 34-cm (blue), and 24-cm (red) pipes. **(E)** Impact force obtained by 10 consecutive trials. **(F)** Radar plots show the relationship among the force, the displacement, the speed, and the duration for each impact. **(G)** Mean values for the force, the displacement, the speed, and the duration. Error bars represent the standard deviation. *p* > 0.05; ^*^p < 0.05; ^**^p < 0.025; ^***^p < 0.01; ^****^p < 0.005; ^*****^p < 0.001.

For Phase 2, the spring-load impactor, we performed a series of impacts (10 impacts for each setting) on surgical gel pads (Oasis Universal Table Pad OA030, Truelife, Dublin, Ireland). The tension adjustment screw was set to a specific number of turns. This number of turns is correlated with the compression distance (Figure 2B) measured with a Vernier caliper (Clas Ohlson, Oslo, Norway). This enables a precise repositioning of the tension screw after dismounting and remounting of the impactor. Because the weight-drop impactor was tested on a difference surface (10 % gelatin) we also performed additional series of impacts on gel pad to have a comparable conditions (Figure 4).

### Identification of the Thoracic Level for Surgery

For Pigs 1–12, we used anatomical landmarks—e.g., the scapula and counting of the ribs to identify the thoracic level. From Pig 13 onwards, we performed preoperative X-ray (SoundEklin eSeries 4336R CIE at 80 kW Carlsbad, CA, USA) and inserted a 22 G needle in the paravertebral musculature as a landmark. Once the level was confirmed, the needle was removed and the skin labeled with a permanent marker.

### Sedation, Anesthesia, and Analgesia

All anesthetic procedures were supervised by a trained veterinary anesthetist. For premedication, the pigs received an intramuscular injection of ketamine 10 mg/kg, midazolam 1.0 mg/kg, and dexmedetomidine 15 μg/kg prior to the catheterization of the auricular vein. After preoxygenation, anesthesia was induced with propofol intravenously to effect. After endotracheal intubation, intermittent positive pressure ventilation was initiated. Anesthesia was maintained with propofol 5–10 mg/kg/h, ketamine 5 mg/kg/h, and dexmedetomidine 4–8 μg/kg/h. Three to five minutes prior to the SCI, a bolus of rocuronium 1.2 mg/kg was injected intravenously to prevent any spontaneous movement. Physiological parameters, including heart rate, ECG, invasive arterial blood pressure, E_T_CO_2_, S_p_O_2_, and body temperature, were monitored using a multi-parameter monitor (GE Carescape 650, GE Healthcare, Finland). In addition, the anesthetic depth was continuously monitored. It should be noted that the temperature at the start of the surgery was 38 ± 0.9. In all the pigs, it rose along with the surgery by 0.98 degrees ± 0.77.

### Intraoperative Electrophysiology

For somatosensory evoked potentials (SSEP) recordings, we stimulated the median and tibial nerves on each side using 13-mm subdermal needle electrodes 27 G, (product# S44–837, Technomed Europe, Beek, The Netherlands). SSEP were acquired from SCI 5–12 and 17–19 with an ISI IOM system portable (INOMED Medizintechnik, Emmendingen, Germany). The cranial electrodes were subdermal needles (29) or screws (24-mm long for 3.5-mm diameter) if the impedance was too high. The screws were connected to the amplifier by crocodile connectors, as previously described (30) and reported for MEP stimulation (31–33). Nerve stimulation was performed at 3.7 Hz, 100 μs pulse duration, and intensity 10–50 mA for 150–200 averages. SSEP were recorded continuously during the whole procedure. In one case (SCI 18), we recorded SSEP in response to epidural stimulation using a monopolar probe (product# 3602-00, Technomed Europe) to stimulate the spinal cord cranial and caudal to the injury. Epidural stimulation was performed at 1 Hz, 200 μs pulse duration, and intensity 3 mA for 50 averages.

### Surgery and Spinal Cord Injury

The surgery was performed by a trained neurosurgeon. The pig was placed in a prone position on the operating table. Under deep anesthesia, a 10-cm posterior midline incision was made between T8 and T12. Mobilization of the paravertebral muscles was performed with electrocautery, while diathermy was used to control haemostasis. The spinous processes, transverse processes, and the laminae of T9-11 were exposed.

For Phase 1 experiments (SCI 1-9), we removed two spinous processes T10 and 11, but the lamina remained intact. The laminae were flattened with a high-speed diamante drill (Surgairtome Two, Hall/Linvatec) to enable the positioning of the lamina plate (Figures 1A,B,F). We removed equal parts of both laminae to create space for the impactor without performing a complete two-level laminectomy (Figure 1I). The lamina plate was positioned to align its opening with the opening in the laminae; it was attached to the remaining laminae parts by inserting 8-mm laminae screws (Figure 1J). A spirit level (Figure 1F) was used to evaluate the horizontality of the surface. The pipe was subsequently attached to the articulated arm (Figure 1B element 6); the plunger that contained the load sensor was inserted at the bottom of the pipe; and the pipe was connected to the opening of the lamina plate (Figure 1A). Because of this positioning, the plunger was lying over the dorsal surface of the dura. The spirit level was introduced at the top opening of the pipe to check its verticality and subsequently replaced by the electromagnet, powered and loaded with the 50-g weight (Figure 1K). To minimize movement, the ventilator was stopped and the impact was initiated by depowering the electromagnet. After the impact, the impactor was disassembled, the lamina plate was removed, and the spinal cord was rinsed with NaCl 0.9%.

**Figure 5:**
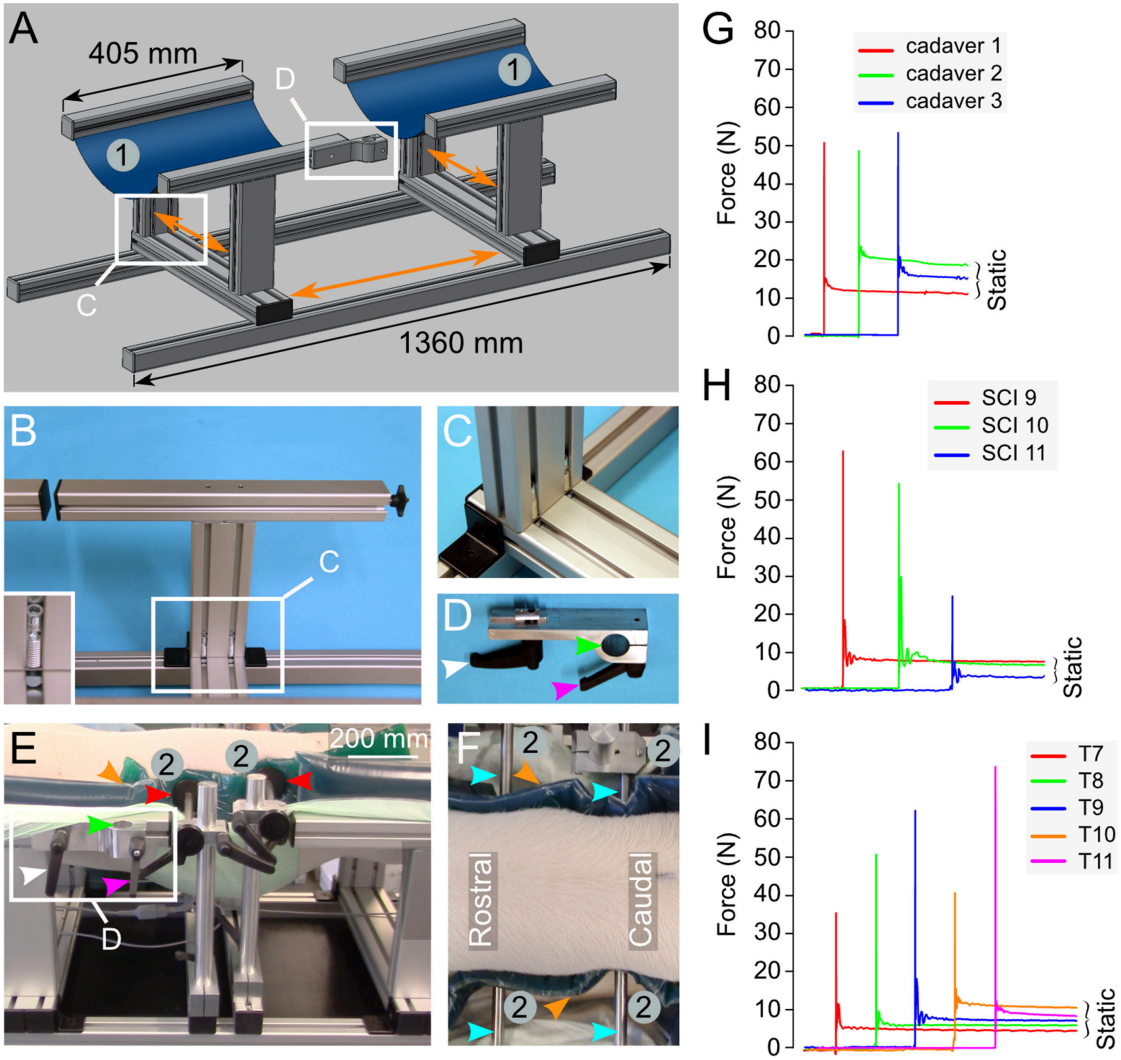
Engineering of a pig-operating table inspired from earlier studies (26, 27). **(A)** Drawing representing the table composed of aluminum parts mounted on rails. This enables the adjustment of the different parts in various directions as shown by the orange arrows. Two large blue rubber bands (1) mounted on the T-shaped elements provide the support to the pig in a prone position. **(B,C)** Details on the T-shape element and its assembly. The inset in **(B)** shows the screw system that attaches the T-shaped element to the rails. **(D)** A binder element is used to attach the articulated arm to the operating table. It is inserted in the side rail of the T-shaped element as shown in **(A,E)**. It can be locked in the desired position using the screw handle (white arrowhead). The rod attached to the articulated arm (Figure 3A) fits the hole of the binder element (green arrow head) and another screw handle (magenta arrowhead) presses on a cleft to reduce the diameter of the hole and immobilize the rod. **(E,F)** A pig placed in the prone position on the table. Two horizontal supports (2) are positioned on the operating table using the same rails and handle screw system as described above. This lateral support system may be moved in the direction of the three body axes. Plastic discs (red arrowheads) are mounted at the tip of the stabilizing rods (cyan arrowheads). They press on dumping gel pads (orange arrowhead) to prevent skin pressure sores. **(G–I)** Force recorded on impact in 3 different pig cadavers **(G)**, 3 different pigs *in vivo* **(H)** and 5 different spinal levels of the same pig *in vivo* **(I)**. All the numbering and colored arrowheads are consistent throughout this figure.

**Figure 6:**
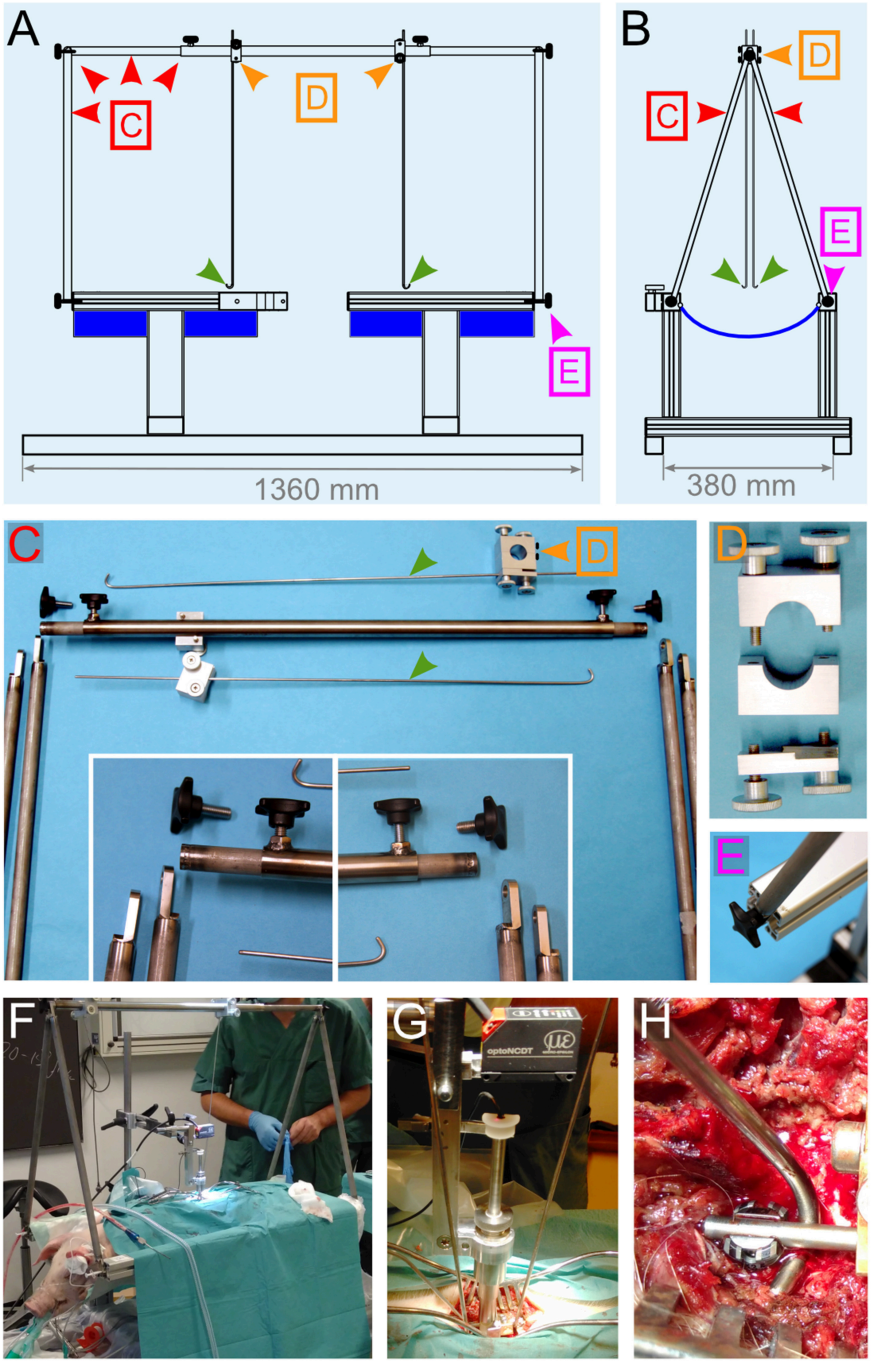
Engineering of a suspension system. **(A,B)** Drawings of different views of the pig-operating table equipped with the suspension system. The lateral support rods depicted in Figures 5E,F were replaced by a stand (red arrowheads) that supports two vertical rods, which get terminated with a hook shape (green arrowheads). The positioning and locking system [orange arrowheads and **(D)**] enables the rods to be moved in the desired position and firmly affixed. The function of these rods is to be attached to the spinal column and pull on it upwards to straighten it. **(C–E)** Details for the different elements that compose the suspension system. **(F–H)** Assembly of the suspension system during surgery. **(F)** Overview of the operation table. **(G)** Close-up on the impactor and the suspension rods. **(H)** Attachment of the hock to the spinal rod. All the colored arrowheads are consistent throughout this figure.

**Figure 7:**
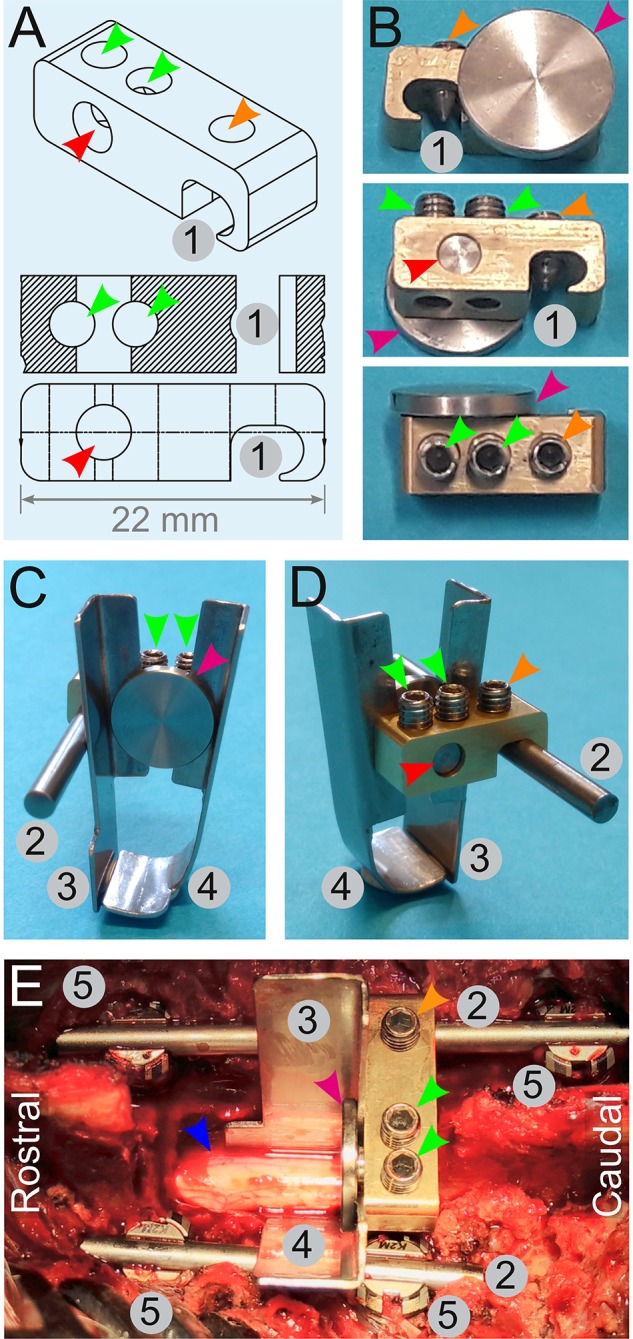
Engineering of a spinal cord support system. **(A,B)** Drawings **(A)** and pictures **(B)** for the engineering of a link element. It is composed of an indentation (1) to affix it onto a rod (2) by a screw (orange arrowheads). Two other screws (green arrowhead) tighten or release a small cylinder (red arrowhead) terminated by a disc (magenta arrowhead). Tightening of these screws (green arrowheads) pulls on the cylinder and presses the disc against the edge of the link element. This is used to affix two plates **(C,D)**—one straight (3), and the other is bended (4). **(E)** Assembly of the spinal cord support system *in vivo*. Four pedicle screws (5) are inserted and two rods (2) are fitted and secured on the screws to fuse three adjacent spinal segments. The link element is attached to the longest spinal rod. The plates (3 and 4) encompassing the spinal cord (blue arrowhead) are firmly pressed against the link element by the disc (magenta arrowhead). All the numbering and colored arrowheads are consistent throughout this figure.

For Phase 2 of our study (SCI 10–20), we developed the spring-load impactor system (Figure 2). This system was first used with lateral external support of the whole pig attached to the surgical table (Figures 5A,E,F). This was adjusted prior to surgery by applying pressure on the horizontal rods. To avoid pressure sores, large discs were mounted at the tip of the rods and gel pads were placed between them and the skin of the pig. Later, in the developmental process, we replaced this external support by internal implants to assemble a vertebral column (Figure 6) and a spinal cord supports (Figure 7).

As previously described, the spinous processes, transverse processes, facet joints, and the laminae T11–13 were exposed. We performed a two-level laminectomy of T12–13 and T11 was partially removed. To gain sufficient lateral space, we removed the medial parts of the facet joints and left the nerve roots untouched. For the vertebral body fusion, we used 25-mm titanium screws and rods designed for human use in the cervical spine (Mesa mini, K2M). With the help of anatomical landmarks, we inserted pedicle screws in T11 and T12 on the left side and in T11 and T13 on the right side of the pig (Figures 7E, **10A–C**). Two titanium rods were attached and secured to the pedicle screws. A link element designed for this purpose (Figures 7A,B) was mounted on the right-side rod. To provide ventral and lateral support, a bended plate (37-mm long, 10-mm wide at the top, and 19-mm wide at the bottom) was inserted underneath and lateral to the spinal cord on the left side. A second straight plate of the same dimensions was placed on the right side, lateral to the spinal cord, until it met the bended part of the first plate underneath the spinal cord (Figures 7C–E). Nerve roots were exposed and untouched during this process. Both plates were fitted between a small disc and the edge of the link element. Tightening of the screws pulled the disc to press the plates against the link element. Suspension rods were hooked to the pedicle rods and used to pull up and straighten the vertebral column (Figures 6F–H). The spring-load impactor was positioned with the tip placed between the two plates. We used the real-time read-out from the load sensor to ascertain that the impactor was touching the dura without compressing it. The tare of the load sensor was zeroed before approaching the dura. As the impactor tip touches the dura, the load value increases slightly. The surgeon further adjusts the position until the read-out is zero Newton and the arm is locked. This is done under visual control of the surgeon. The ventilator was stopped, and the release of the piston was triggered by pulling the release mechanism (Figure 3B). One minute after the impact, ventilation was resumed and the compression was maintained for four extra minutes for a total of 5 min inspired from Lee et al. (22). The suspension rods, the spinal plates, and the link element were dismounted, while the spinal cord was rinsed with NaCl 0.9%.

### Postoperative Imaging

For *in vivo* MRI scans (SCI 1–7), an active drain (FG 14 Exudrain, Wellspect HealthCare, Sweden) was positioned, haemostasis was checked, and the muscle and skin were sutured using Monosyn sutures (B Braun, Vestskogen, Norway). The pig was disconnected from the automated ventilator and connected to a manual ventilation system for its transportation to the MRI facility. Anesthesia was maintained by propofol in combination with ketamine and vital parameters were monitored. T1 and T2-weighted images were acquired with a 3 T Philips MR Imaging DD 005. We also performed two CT scans (BrightSpeed S system) to confirm the laminectomy level and the screw positioning (SCI 18 and 19).

### Dissection of the Spinal Cord and Termination

While the pig was deeply anesthetized, the laminectomy was extended to four levels. A neuromuscular blocker (Rocuronium 1.2 mg/kg) was injected (i.v.) to prevent any uncontrolled movement. The thoracic roots were inspected and no macroscopic damage was detected. Furthermore, the roots were cut and the spinal cord was transected at the rostral and caudal borders of the laminectomy. It was subsequently immersed in ice-cold PBS and maintained at 4°C until further dissection and tissue fixation were performed. The pig was euthanized by an i.v. injection of potassium chloride.

### Post-mortem MRI

The dura was removed and all rootlets were cut. The spinal cord was immersion fixed in 4% (w/v) paraformaldehyde-PBS for 5 days at 4°C. Two to four days prior to high-resolution MRI scanning, the spinal cord was rinsed in PBS, incubated in 1% (v/v) gadolinium (OMNISCAN, GE Healthcare) diluted in PBS, and maintained at 4°C, as previously reported for non-human primate spinal cords (34). Prior to scanning, the spinal cord (SCI 14-17) was immersed in a glass jar that contained MRI-compatible perfluoropolyether oil (Fomblin, Sigma-Aldrich, 317950-100G). MRI scans were performed on a 9.4 Tesla Agilent small animal MRI system (Agilent Technologies, Palo Alto, CA, USA) using a quadrature radio frequency volume coil (19-mm inner diameter, Rapid Biomedical, Rimpar, Germany). High-resolution axial and longitudinal images were obtained with a 3D spoiled gradient echo (field of view: 8 × 8 × 28.8 mm, matrix dimension: 256 × 256 × 128). The echo time was 3.3 ms; the repetition time was 20 ms; and the total scanning was 4 h 30 min. Images were processed with ImageJ, while the 3D rendering and calculations of volumes were performed using Free-d (35) (http://free-d.versailles.inra.fr). For quantifying the volume of bleeding, MRI images were converted to 8-bits images and the threshold (0–60) was applied before pixel counting was done.

### Luxol Fast Blue Staining

For histology, the spinal cord was embedded in paraffin (SCI 14, 15, and 17), sectioned at 5–7 μm, mounted on positively charged slides, and then, processed for labeling with 0.1% (w/v) luxol fast blue diluted in 95% ethanol and 0.5% (v/v) glacial acetic acid for 2 h at 60°C. The differentiation was performed using 0.05% (w/v) of lithium carbonate, freshly dissolved in water, followed by a step in 70% ethanol. The sections were rinsed in water, incubated in absolute ethanol and xylene and coverslipped using Eukitt (Sigma-Aldrich).

### Statistics

The data presented indicate mean values (μ) with error bars that represent the standard deviation (σ). In the text, the coefficient of variation (CV) given as a percentage: CV = (σ/μ) × 100 is used to assess the dispersion of the data. Statistical comparison of the mean was done with the Mann–Whitney *U*-test (significance at *p* < 0.05). The calculations were performed using Libreoffice Calc (https://www.libreoffice.org/) or OriginPro 9.2 (OriginLab, Northampton, MA, USA).

## Results

### Phase 1: Development of a Weight-Drop Impactor

#### Testing on Inert Surface

The system was tested on 10% gelatin because its surface has similar properties as the spinal cord. Such properties include consistency and deformation. However, there are several difficulties in using gelatin. If the layer is too thin, the plunger perforates the gelatin and hits the bottom of the dish. Also, the force value recorded is too high and unreliable. Thus, it is necessary to use a thick layer. However, the thickness also determines the amount of energy absorbed. It is, therefore, crucial to use a standard thickness for all impacts. In addition, a specific area of gelatin and its surrounding area may only be used once because it is damaged by the impact.

Repeated impacts on gelatin showed reproducible impact forces specific to the height of the pipe (*p* < 0.001, Figure 1G). The mean impact forces produced by the 44, 34, and 24-cm pipes were 26.3, 21, and 17 N, respectively. The difference between these mean impact values was statistically significant at *p* < 0.001. In line with this, the CV were low, with 6.3% for the 44-cm, 5.4% for the 34-cm, and 4.8% for the 24-cm pipes. In contrast, the values for the displacement, speed, and duration data were relatively close to each other and in some case overlapped between the different pipes. When comparing these values for the 44 and 34-cm pipes or for the 34 and 24-cm pipes, there was no significant difference (*p* > 0.05). However, there was a significant difference (*p* < 0.01) between the displacement values from the 24-cm (5.8 mm, CV 3.4%) and 44-cm (6.7 mm, CV 5%) pipes. Similar results were obtained for the impact speed for the 24-cm (0.57 m/s, CV 15.6%) and 44-cm pipes (0.84 m/s, CV 7.3%). These mean speed values were significantly different at *p* < 0.001. The mean impact duration for the 24-cm pipe was 10.5 ms and CV 16.8%; it was 7.9 ms and CV 5.3% for the 44-cm pipe. These mean speed values were significantly different at *p* < 0.05. A radar plot for each impact shows the relationship among the force, displacement, speed, and duration (Figure 1H). Two clearly separated domains corresponding to 44 cm (green) and 24 cm (red) impacts exist. In contrast, the domain of the 34-cm impact (blue) is not entirely separated and invades the two other domains. Hence, this system generates reproducible impacts on gelatin. However, the 34-cm pipe produced impacts with characteristics that are too close to those of the 24 and 44-cm pipes. Therefore, we predominately used the 24 and 44-cm pipes for further tests *in vivo*.

#### Testing in Pigs *in vivo*

We performed impacts in nine pigs mounting the system, as described in the methods and as shown in Figures 1I–K. Four impacts were performed with the 44-cm pipe and the peak impact forces were 20.2, 29.5, 29.4, and 57.3 N (Figure 1L). The mean impact force for this group was 34 N and the CV was 47%—this indicates substantial variability (Table 1). We also performed injuries on four other pigs using the 24-cm pipes. We recorded the peak impact force at 19.1, 12, 5.2, and 27.6 N. The mean for these impacts was 16 N and the CV 60%—this further demonstrates a substantial variability (Table 1). We also injured a single pig with the 34-cm pipe. The peak force recorded, 20.4 N, was in between the means obtained with the two other pipes. However, the variability of the impact force in each group is such that 20 N could also be obtained by any of the other pipes. Overall, such data shows that this impactor generated reproducible impacts on gelatin. However, in our opinion, the variation of the peak impact force *in vivo* was too high. Therefore, we abandoned our prototype in spite of knowing that other groups have obtained reproducible impacts by using other designs of weight-drop impactors (8, 18–22).

**Table 1:** Impact characteristics in different experimental groups.

Phase 1	**PIPE: 44cm**										
										**Mean**	**CV (%)**
	SCI #	1	2	3	4						
	Force (N)	20.2	29.5	29.4	57.3					34.1	47.1
	**PIPE: 24cm**										
										**Mean**	**CV (%)**
	SCI #	5	6	7	8						
	Force (N)	19.1	5.2	12	27.6					16	60.1
Phase 2	**SPRING-LOAD** **+** **EXTERNAL LATERAL SUPPORT**										
										**Mean**	**CV (%)**
	SCI #	9	10	11							
	Force (N)	62.4	53.8	23.2						46.5	44.3
	**SPRING-LOAD** **+** **SPINAL CORD SUPPORT** **+** **FUSION** **+** **SUSPENSION OF VERTEBRAL COLUMN**										
										**Mean**	**CV (%)**
	SCI #	12	13	14	15	16	17	18	19		
	Force (N)	49.8	39.6	44.2	53.6	41.5	42.5	50.8	45.1	45.9	10.8
	Displacement (mm)	7.1	6.5	7	9.2	6.2	7.2	6.9	7.4	7.2	12.4
	Speed (m/s)	1.4	1.2	1.8	1.7	1.5	1.4	1.4	1	1.4	18.2
	Duration (ms)	8.5	8.8	9	10	10.3	8.8	8.5	8.5	9	7.8
	Static force (N)	17.6	20.2	18.6	10.8	15.6	16.5	14.3	14.5	16	18.1

*Note that one additional pig was injured with the 34-cm pipe resulting in a 20.4 N impact*.

### Phase 2: The Spring-Load Impactor

Besides variation, during Phase 1, we identified a number of limitations such as visual control and use of a fast camera or sensor to characterize the biomechanical properties of the impact. Most importantly, we realized that it would be an advantage to gain better control over the static compression. Hence, it was necessary to design a system that would keep the load in position and still exert a certain force. A spring was a simple tool to resolve this problem (Figures 2, 3).

#### Testing on Inert Surface

We performed a series of impacts on two layers of gel pads that were originally used to prevent pressure sores during long-lasting surgeries in humans. As for the spinal cord and gelatin, gel pads have a relatively soft surface and absorb the energy delivered by the impact. However, in contrast to the spinal cord or gelatin, they are not damaged by the impact and do not lose firmness upon repeated trials. It was, therefore, possible to impact the same area of the pad *ad libitum* without affecting the impact characteristics. We performed trials of 10 impacts using 12 different settings of the tension adjustment screw (Figure 4). Each trial showed a highly reproducible impact as characterized by force, speed, displacement, static compression, and static force. For example, at the lowest screw setting, the average peak impact was 5.56 N with almost negligible variations (CV 1.9%) (Figure 4A). Starting with 12 screw turns, a decrease in the number of turns proportionally reduced the peak impact force and the static force (Figure 4B). A radar plot that links the values of all impact parameters for each of the 120 impacts shows well-separated domains for different screw settings (Figure 4C). In general, plotting the mean values from the different impact parameters measured against the number of turns showed a nearly linear relationship (Figure 4D). In addition we performed a series of impacts with the weigh-drop impactor on gel pad so that both impactors are tested with similar conditions (Figures 4E–G). This resulted in impact force characterized by a small variation (1.3% for the 24-cm, 2% for the 34-cm, and 2.8% for the 44-cm pipes). In contrast, the values for the displacement, speed, and duration were relatively close to each other and in some case overlapped between the different pipes to the point that the there was no significant difference in impact speed for any pipes. The radar plots shows clear color domains for the force and the displacement but not for the speed and the duration. Thus, such data shows that our spring-load impactor delivers reproducible impacts on gel pads with superior characteristics than our weight-drop impactor. In addition, the spring-load impactor can be adjusted to obtain different impact strengths.

#### Testing in Pigs *in vivo*

We encountered several surgical difficulties with the pig in a prone position on a flat operating table. Haemostasis was affected because of the increase in the venous pressure because of the prone position. Another difficulty included the attachment of equipment such as the articulated arm for the impactor. Hence, inspired by earlier reports (26, 27), we developed a pig operation table (Figure 5), as described in the methods. We first tested both the table and the spring-load impactor on three pig cadavers. The results showed three nearly identical peak impact forces at 50.9, 48.8, and 53.5 N (CV 4.6%) and a closely related static compression (Figure 5G). We further performed three similar tests *in vivo*. From the first two pigs, we obtained similar peak impact forces at 62.4 and 53.8 N with identical static compressions at 6.9 and 7 N (Figure 5H). However, the peak impact force on the third pig was substantially lower at 23.2 N with a lower static compression at 2.8 N. The CV for the peak force in this group was 44.3%, indicating a substantial variation (Table 1). These results lead us to think that the impact force could be related to the vertebral levels. We performed further impacts *in vivo* in a single pig at different vertebral levels from T7 to T11 (Figure 5I) and obtained a mean impact force at 52 N with a CV at 30.8%. However, there was no clear relationship between the impact strength and the rostro-caudal direction with 35.5 N at T7, 48.5 N at T8, 62.4 N at T9, 39.4 N at T10, and 73.9 N at T11. This finding suggests that the problem was related to the impact mechanism and potentially the deformation of the spinal cord or the surrounding tissue. In addition, we suspected that the spinal cord could eventually “escape” the impact by sliding aside.

#### Additional Support Systems

We, therefore, decided to rigidify the vertebral column via the fusion of three adjacent vertebral segments. In addition, we modified the pig-operating table to implement a suspension system to straighten the vertebral column (Figure 6). We also introduced a spinal cord support system to enclose the ventral and lateral parts of the spinal cord (Figure 7).

**Figure 8:**
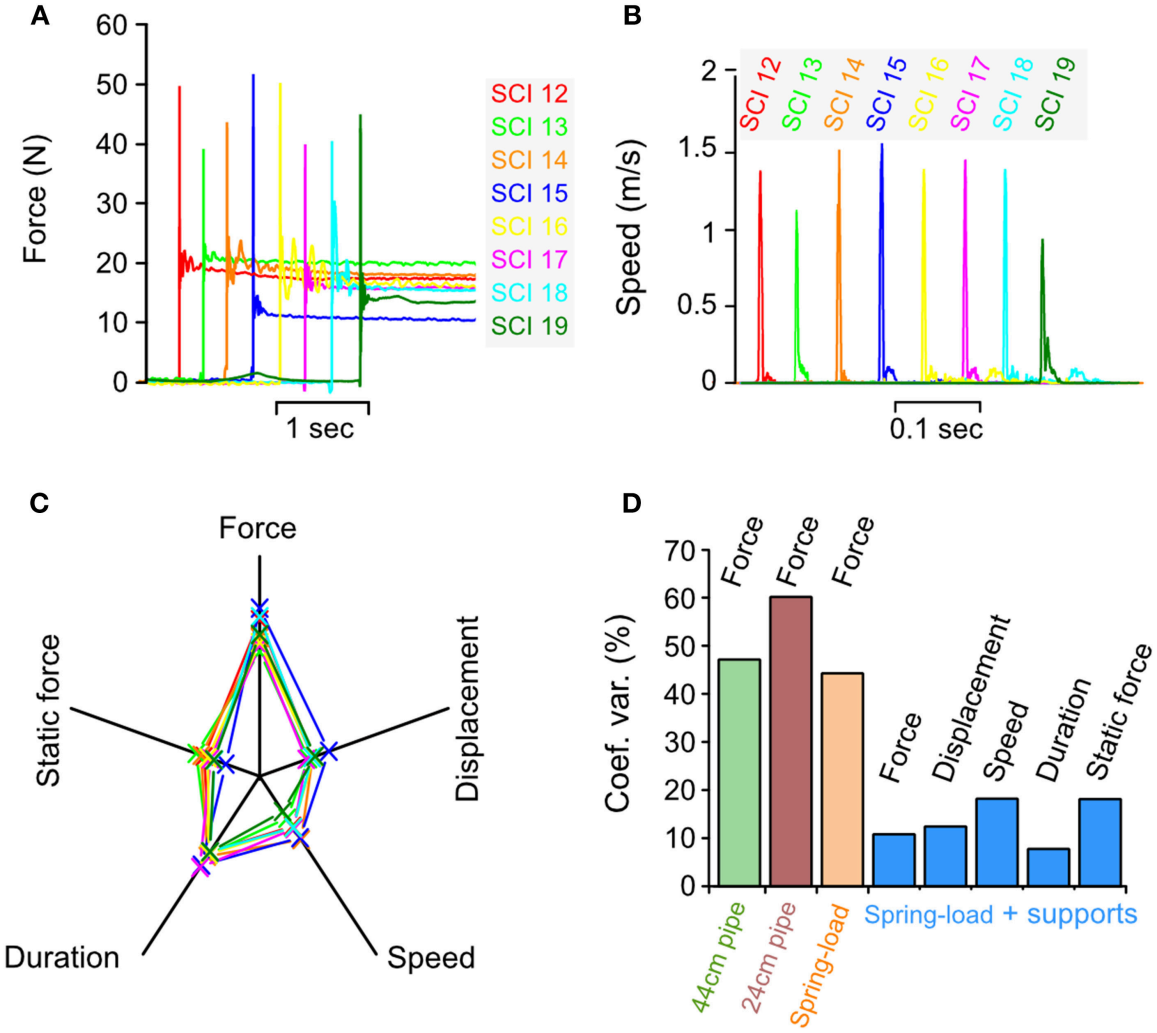
Biomechanical characterization of impacts *in vivo*. Parameters recorded during an impact in 8 different pigs using spring-load impactor, the spinal cord support and the vertebral column suspension systems. **(A)** Impact force. **(B)** Impact speed. **(C)** Radar plots showing the relationship among the force, the displacement, the speed, the duration, and the static force for each impact. **(D)** Comparison of the coefficient of variation for impact parameters recorded during weigh-drop (44 and 24-cm pipe), spring-load, and spring-load with supports.

We tested these changes *in vivo* on eight pigs with the same setting for the tension adjustment screw. We obtained eight reproducible impact forces in both the dynamic and static phases (Figure 8A and Table 1). The average peak force was 45.9 N and the CV at 10.8% only exhibited a small variation. The same finding was obtained for all other impact parameters for which the CV was between 7.7 and 18.2%—this indicates a high level of inter-impact reproducibility (Figure 8 and Table 1). The radar plot exhibits the same pattern for all impacts (Figure 8C). Comparison of the CV of all three systems shows a clear improvement of the reproducibility in favor of the spring-load impactor combined with the support systems (Figure 8D).

#### Loss of SSEP After Spinal Cord Injury

**Figure 9:**
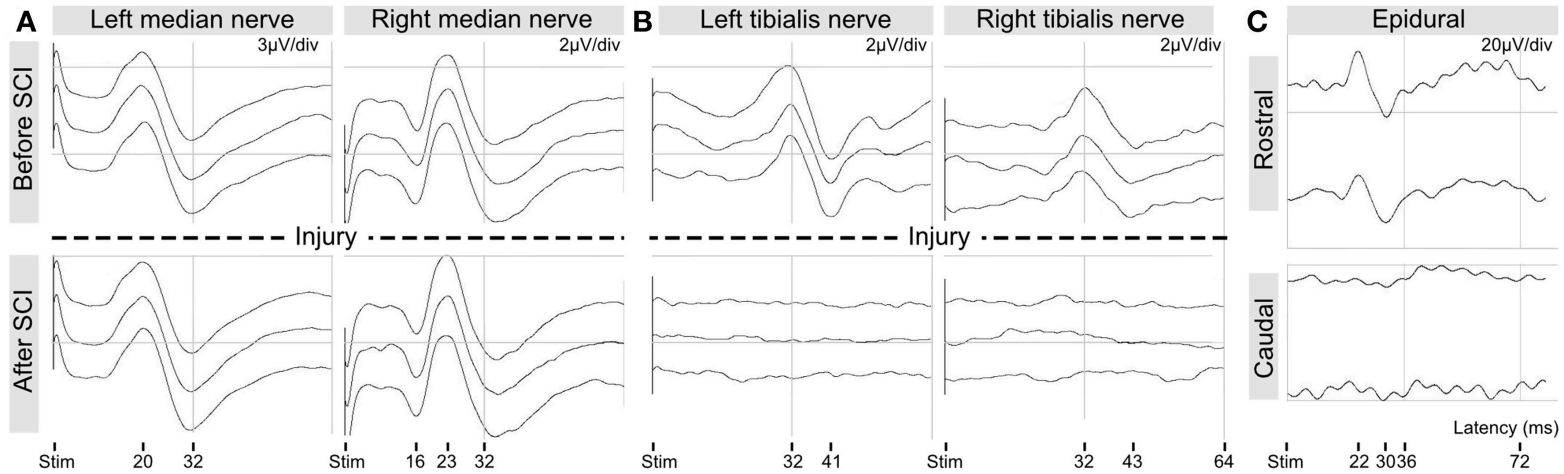
Intra-operative recordings of somato-sensory evoked potentials. **(A)** SSEP from median nerve stimulation. **(B)** SSEP from tibialis nerve stimulation. **(C)** SSEP from epidural spinal stimulation rostral and caudal to the injury.

SSEP are commonly used to assess the integrity of the spinal cord in both clinical and animal research (30). In this study, we continuously recorded SSEP during the whole procedure (Figure 9). SSEP were not affected by the surgical procedure prior to impact. The SSEP elicited from the median nerves (left and right) were not affected by the impact because they reflect signals transiting rostral to the injury (Figure 9A). In contrast, the tibialis nerve SSEP were always lost immediately after impact and did not recover for the remaining time (~1 h) of the surgery (Figure 9B). In one pig, we also recorded the SSEP elicited by an epidural stimulation of the spinal cord (Figure 9C). Epidural SSEP were obtained when the monopolar stimulation electrode was positioned rostral to the injury. In contrast, no epidural SSEP were obtained when the electrode was positioned caudal to the injury. Here our electrophysiological recordings suggest that the impacts resulted in severe functional losses.

#### Postoperative Radiology

**Figure 10:**
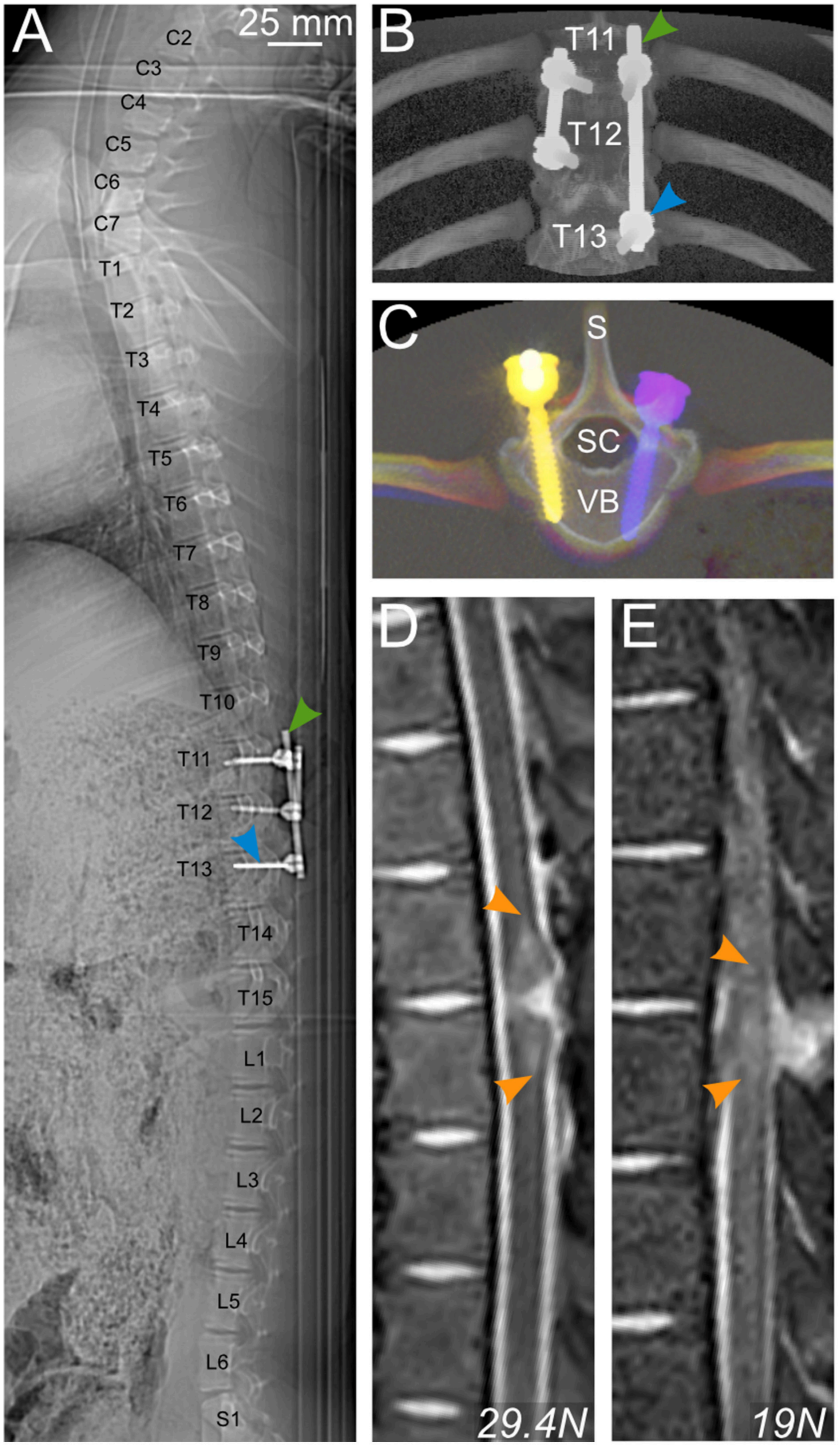
Postoperative radiology. **(A–C)** CT scan of a pig spine from C2 to S1 **(A)** and 3D rendering **(B,C)** enable control of the positioning of pedicle screws (blue arrowhead) and rods (green arrowhead) for bilateral fusion of T11–T13 and T11–12. **(D,E)** T2-weighted MRI scans showing a longitudinal view of the spinal cord and the vertebral column of 2 pigs injured with different impact strengths. Hyperintense signal in the spinal cord is caused by bleeding. S, spinal process; SC, spinal canal; VB, vertebral body.

CT scans were performed to evaluate the positioning of the implants (Figures 10A–C). In these images, the link element is not visible because we removed it, lest it creates too much artifacts and degrade the quality of the pictures. However, it was positioned at T12, attached to the right rod (Figure 10B), and thus confirmed that the injury was between T11 and T12, as targeted by preoperative X-rays. The pedicle screws were introduced to the vertebral body with an angle of 8–16 degrees, leaving the spinal canal untouched (Figure 10C). We also performed *in vivo* MRI scans to evaluate the strength of the injury (Figures 10D,E). T2-weighted images showed an intra-spinal inflammation halo, whose size correlated well with the injury peak force recorded during the impact. It is to be noted that the hypertensive signal is a result of bleeding.

#### The Impact Produces Extensive Tissue Damage

**Figure 11:**
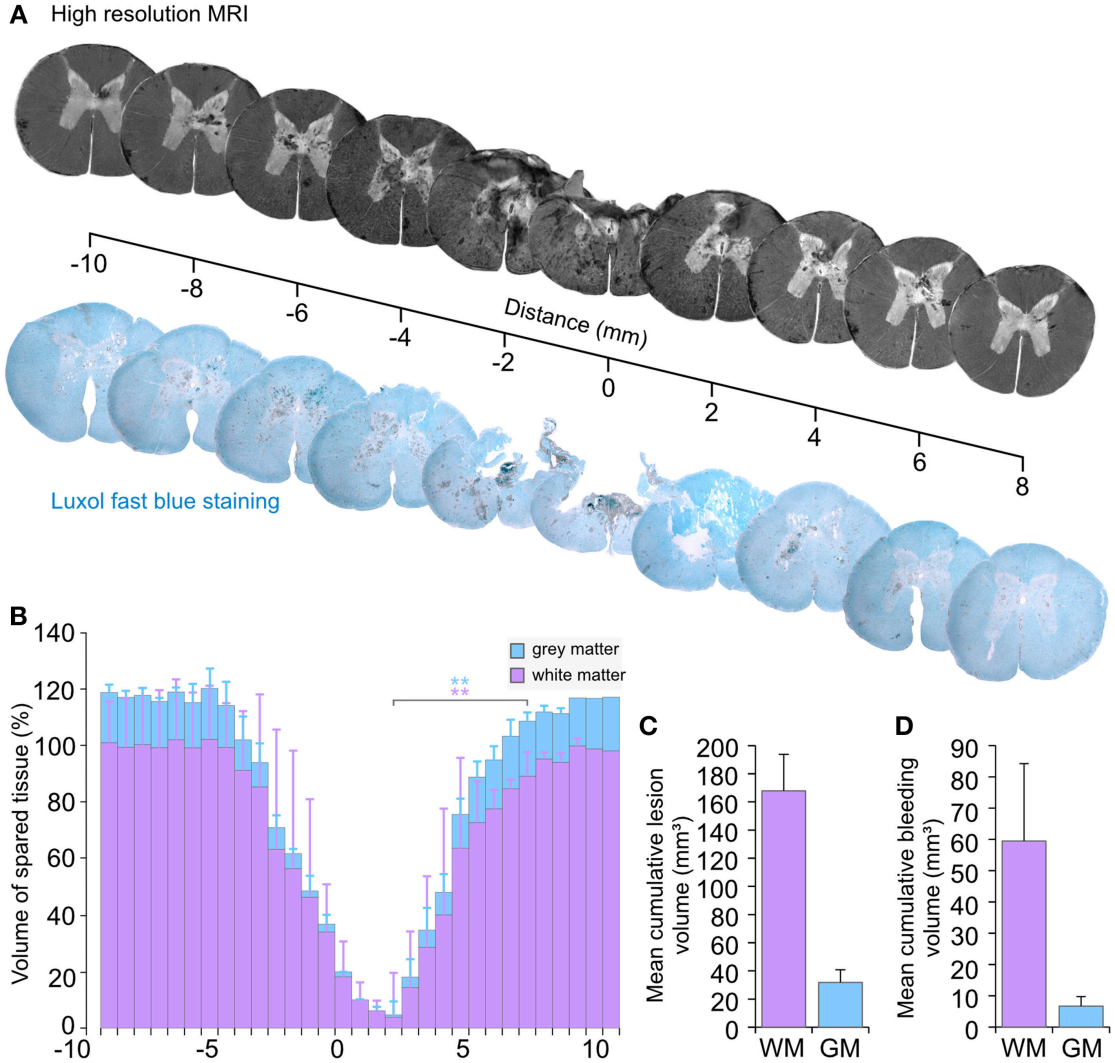
Tissue damage following an injury by the spring-load impactor. **(A)** Axial views from high-resolution T1-weighted MRI compared with luxol fast blue staining of histological sections. **(B)** Volume of spared spinal tissue. **(C)** Cumulative lesion volume. **(D)** Cumulative bleeding volume. GM, gray matter; WM, white matter. ^**^p < 0.025.

Post-mortem high-resolution 9.4 T MRI (lateral resolution 31.2 μm) scans (SCI 14–17) indicated a severe and broad damage area in which tissue destruction coexists with multiple intramedullary bleedings (Figure 11A). This finding was further confirmed by the staining of histology sections with luxol fast blue. In line with other reports, we found that high-resolution MRI was consistent with histology (34, 36). However, sectioning the tissue through the epicenter of a newly damaged spinal cord can result in loss of parts or the entire section. Therefore, we performed the volume analysis on high-resolution MRI images. Interestingly, this type of experiment has also been performed in SCI pigs in a recently published study (37). Our results showed a decrease in the intact white and gray matter volumes at the level of the injury (Figure 11B). In MRI slices from the undamaged spinal cord tissue, the mean gray matter volume was 6 mm^3^ (CV 17.3%). For convenience, we defined this value to be 100% and expressed further volume values in Figure 11B as percentage. Hence, the white matter volume from this undamaged slice was 17% (CV 13.2%). A significant (*p* < 0.025) reduction in these volumes was identified at the epicenter of the injury with a volume of white matter at 10% (CV 126%) and a volume of gray matter at 0.05% (CV 125.7%). At the epicenter, the CV exhibited substantial variations between the different samples. However, these volumes are so small compared to the uninjured tissue that such variations are almost irrelevant.

The mean cumulative tissue loss (Figure 11C) for the white matter was 167.8 mm^3^ with a CV that represented a variation of 15.4%. The cumulative loss of the gray matter was 31.8 mm^3^ with CV at 28.2%.

Substantial intra-spinal bleeding is observed not only at the injury area, but also in the perilesional zone (Figure 11A). Pixel analyses of MRI images were used to quantify the volume of bleeding. The mean cumulative volume of the bleeding presents a variation with a CV at 41.6% of the mean for the white matter and 45.5% for the gray matter (Figure 11D). Overall, such data confirms a consistent loss of the white and gray matter and a severe injury of the spinal cord, which combines tissue loss with bleedings that extend beyond the injury area.

## Discussion

### Impactor Design

Several types of SCI have been experimented in pigs and may be categorized as follows: (1) compression injuries using clips (38–40) or computer-controlled stepping motor (41), (2) ischemic injuries with aortic cross or segmental artery occlusion (42–44), (3) root avulsion with ventral myelotomy (45), (4) vertebral column distraction/retraction (46–48), (5) spinal cord transection/hemisection (49–51), (6) spinal cord contusion using the controlled cortical impactor (29, 37, 52) or a weight-drop impactor (18–23). Despite these studies, there are no commercially available devices adapted to the size and the strength required for a 20–50 kg animal. This potentially hinders SCI researchers to use large animals because it requires, as a first step, the engineering of a reliable impacting device. In this report, we detailed the methods and provided all necessary information to duplicate the apparatus we created.

Weight-drop contusion devices have been widely used to generate graded and reproducible injuries in different animal models (8, 18–22). We, therefore, initially engineered a weight-drop impactor. However, our design led to disappointing results and non-reproducible impacts *in vivo*. We encountered several issues. Variations in the thickness and wideness of the lamina created problems in positioning the lamina plate. Even with different plate sizes, the variation in the distance between the lamina and the dura was a recurrent problem to place the plunger in a proper position (midline and surface of dura). Hence, our concept of the lamina plate that was intended to rigidify the vertebral column and guide the plunger was riddled with difficulties. This was worsened by the opacity of the metallic pipe which denies visual control over the plunger. It also created difficulties to record some of the impact biomechanical parameters with a high-speed camera. An attempt to use a transparent plastic pipe was not successful because of frictions, which influenced the impact reproducibility. A glass pipe might have solved this issue. However, working with glass presented other problems for our local workshop and therefore this idea was abandoned. However, the weaknesses of this system included the reproducibility of the impact (discussed below) and the implementation of a static compression, following the initial impact to mimic traumatic SCI in a better way. Lee et al. (22) resolved this problem by placing a 100-g load on top of the 50-g load initially used for the dynamic impact. This was possible because of an open rail system, but it was impossible to standardize the implementation of this strategy in a reproducible manner in our closed pipe.

We engineered a new apparatus, which represents a different and, to our knowledge, a novel system. The requirements for this system were: (1) easy handling (2) reproducible impacts, (3) adjustable impact strength, (4) the possibility to apply a static compression following the initial impact, (5) better characterization of the impact with more sensor feedback, and (6) visual control for the positioning of the impactor tip on the dura. The pipe and the weight were replaced by a piston system driven by the mechanical power of an expansion spring. This approach presented multiple advantages: It is a one-piece impactor, which is easier to handle. Moreover, it does not require any assembly during the surgical procedure. Nothing prevents the visual control of the position of the piston tip on the surface of the dura. The system is suitable for use with a high-speed camera. Although for the sake of simplicity and precision, we preferred a laser displacement sensor. In addition, the spring-driven impactor can produce multiple grades of injury strength because of the adjustment screw. This is in contrast to the weight-drop system in which the impact strength depends on the length of the pipe. This was a restriction on the choice of the injury grade. An additional advantage of the spring-load impactor is that it provides a simple solution to combine a dynamic impact with a prolonged static compression. This static compression may be adjusted to a desired force or displacement after the impact. It is also possible to perform a sole stepwise spinal cord compression injury by positioning the impactor on the dura and adjusting the screw until the desired compression force or displacement is obtained. In addition, it is possible to vary the duration of the static compression to influence the injury severity and the balance between the contusion and compression components. It is also possible to mix strong contusion with weak static compression or vice versa. This versatility is an advantage for testing the robustness of a treatment. Initially, it is natural to study a defined experimental treatment with a defined injury characteristic. However, another important step would be to test the treatment in a variety of situations to characterize its indication/limits. For example, it has been suggested that the neuroprotection provided by hypothermia is more pronounced for compression with a certain degree of ischemia than for contusion injuries (53–55). This could be tested using our impactor set for contusion with almost no compression vs. no contusion but slow and prolonged compression.

Thus, the functionality and versatility of this spring-load impactor makes it an excellent tool to generate different graded SCI in large animal models with characteristics superior to those of our original weight-drop impactor.

### Reproducibility of the Impact

In this study, the main challenge was to obtain a reproducible impact *in vivo*. This is critical for SCI models, particularly for demonstrating the efficacy of a preclinical treatment. In addition, a high degree of reproducibility contributes to animal welfare because it reduces the number of animals necessary for significant findings. Furthermore, experiments performed in large animals are long, expensive, and require more human resources. It is, therefore, difficult to compensate for varying degrees of injury with a larger number of animals. In line with a recent study that shows substantial differences between *in vivo* and *ex vivo* impact characteristics (56), we experienced a remarkable difference in the reproducibility of impacts on an artificial surface and *in vivo*. One reason for this finding may be anatomical variation. So, a first step toward standardization was the introduction of a preoperative X-ray, instead of anatomical landmarks, to perform the surgery at the same level. We did not have the possibility to perform intraoperative X-rays. This is preferable as it is more precise. To avoid intra-operative artifacts on the X-rays due to the metallic parts of the pig operating table, we plan to replace the T-shaped elements of the pig operating table (Figure 5B) with identical plastic elements (PEEK, can be autoclaved). The introduction of this table fulfills several important functions such as decreasing the venous pressure. It mimics the prone positioning of humans in spinal surgery. We observed less epidural bleeding during the surgical approach. In addition, it provides an attachment for the articulated arm and the suspension system. Although the use of this table may not have a direct effect on the biomechanical reproducibility of the impact, it eases the whole procedure at a low cost.

Another factor that may contribute to the variations in impact strengths is the resistance of the surrounding tissue. During our test series on artificial surfaces, we determined that any impact on the soft material has a lower peak force value than any impact on the hard material because of a higher absorption of energy. Therefore, we had to consider that anatomical variations (i.e., the size of the spinal canal, disc space, CSF, and venous plexus) may influence the impact strength. A variable is the cushion effect of the CSF. Pigs and other larger mammals have a larger CSF space than rodents (22, 57). Furthermore, pigs have large epidural veins, and their venous plexus is located lateral and ventral to the dura. The dilatation of these veins depends on several factors like positioning, circulation, and hydration status. This contributes to variations in the pressure in the spinal canal and the CSF. This may be difficult to control. We focused on the standardization of the resistance provided by the surroundings of the spinal cord. In our postoperative MRI scans, we determined that the injury site was close to the intervertebral disc in some cases (Figure 10D). The disc represents a softer surface than the bone of the vertebral body. Therefore, any impacts close to the disc would have a different force than the ones in the middle of the vertebral body. To address these issues, we developed a device to enclose the spinal cord with metallic plates. This approach provides the same resistance around the spinal cord for each experiment. This also prevents the spinal cord from sliding aside during the impact. Another potential source of variation is the flexion of the spinal column in response to the impact. This problem was previously identified in rodent experiments and solved by sliding metallic stabilizers under the transverse process or using spinous clamps (9, 58–62). In non-human primates, the spinous processes were clamped and pulled upwards to obtain a more rigid column (57). In our study, we fused three neighboring vertebrae to avoid vertebral bodies to yield at the impact level. In addition, we attached a suspension system to the titanium rods to pull upward and straighten the vertebral column. This eases the positioning of the impactor at a right angle to the spinal cord and it decreases breathing artifacts during the 4 min of static compression done with ventilation. This does not influence the complexity of the procedure as it is easy to handle and can be produced at a low cost. With this approach, we achieved a completely rigid spinal column.

Overall, we showed that these standardization efforts were successful, although they globally increased the complexity of the procedure. However, for experimentations on a large animal model for spinal cord injury it is necessary to work with a trained surgeon. It can be a neurosurgeon, an orthopedic surgeon, a trauma surgeon, and a veterinarian trained in spine surgery. In these circumstances, the additional steps we presented do not present any obstacle.

### Toward a Completely Characterized Large Animal Model

This study, describes the engineering of an apparatus and the establishment of surgical procedures that result in the generation of reproducible spinal cord impacts *in vivo*. It is the first step toward the establishment of a complete animal model. The characterization of the biomechanical properties of the impact provides important information about the generation of the injury. In addition, SSEP and MRI are useful tools that, together with the impact properties, present a comprehensible initial characterization of the injury. However, they only show a snapshot of the situation, and the development of the secondary injury will likely aggravate the functional outcome. Therefore, further experiments on chronic animals using different longitudinal outcome measurements, such as motor behavior, electromyogram recordings, and the study of autonomic dysfunction, will be necessary to obtain a full characterization of the model. We will also need to evaluate the stability of the column because of the surgical procedure. It is possible that we would have to leave the implants to avoid kyphosis in a chronic animal. In fact, this may present the advantage to better model the situation of traumatic SCI in humans. However, such a study is beyond the scope of this paper that only focuses on the development of tools and surgical procedures to generate injuries that are as reproducible as possible. However, this may be the most important step toward the generation of a reliable model. The injury we reported here is strong. With the development of the secondary injury process, it is possible that the severity of this trauma will be beyond any possible repair. So, further experiments will also be necessary to identify the appropriate injury strength that can be used for assessing treatment effects. This will be possible because our impactor can be adjusted to provide multiple injury strength.

## Author Contributions

MZ and J-LB initiated the study, designed the experiments, and designed the different apparatus. MZ performed the surgical procedures. AL, VB, and HH established and performed the anesthesia procedures and the monitoring of vital parameters. EK and J-LB performed intra-operative electrophysiology. LZ and J-LB performed MRI scans. MZ and J-LB wrote the paper, prepared the figures, and revised critically manuscript. All authors read and approved the final manuscript.

### Conflict of Interest Statement

The authors declare that the research was conducted in the absence of any commercial or financial relationships that could be construed as a potential conflict of interest.
